# Single Crystalline Ultrathin Nickel–Cobalt Alloy Nanosheets Array for Direct Hydrazine Fuel Cells

**DOI:** 10.1002/advs.201600179

**Published:** 2016-12-20

**Authors:** Guang Feng, Yun Kuang, Pengsong Li, Nana Han, Ming Sun, Guoxin Zhang, Xiaoming Sun

**Affiliations:** ^1^ State Key Laboratory of Chemical Resource EngineeringCollege of ScienceBeijing University of Chemical TechnologyBeijing100029P. R. China; ^2^ State Key Laboratory of Chemical Resource EngineeringCollege of EnergyBeijing Advanced Innovation Centre for Soft Matter Science and EngineeringBeijing University of Chemical TechnologyBeijing100029P. R. China

**Keywords:** direct hydrazine fuel cells, nickel–cobalt alloys, single crystalline, ultrathin nanosheets

## Abstract

**Ultrathin 2D metal alloy nanomaterials** have great potential applications but their controlled syntheses are limited to few noble metal based systems. Herein Ni*_x_*Co_1−_ *_x_* alloy nanosheets with ultrathin (sub‐3 nm) single‐crystalline 2D structure are synthesized through a topochemical reduction method. Moreover, the optimized composition Ni_0.6_Co_0.4_ alloy nanosheets array exhibits excellent performances for hydrazine oxidation reaction and direct hydrazine fuel cells.

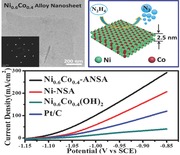

Ultrathin 2D nanomaterials, like graphene or MoS_2_, have garnered tremendous attentions due to their unique properties and great potential applications in various fields.^1^ Especially, ultrathin metal nanosheets exhibit high surface‐area‐to‐volume ratio, high electron mobility, and extraordinary quantum size and surface effects.^2^, ^3^, ^4^, ^5^, ^6^, ^7^, ^8^, ^9^ For instance, the atomic ultrathin Rh,^2^, ^3^ Pd,^4^, ^5^ or Co^9^ nanosheets have shown significant enhancement in catalytic or electrocatalytic properties. Compared to monometallic nanocrystals, alloy nanocrystals have greater potential because their tunable compositions endow them tunable electronic structures and properties, and possible synergistic effect between metals leaving large room for improving the properties like catalysis.^10^ Typically, for the anodic hydrazine oxidation reaction in direct hydrazine fuel cells (DHFCs), NiCo alloy has demonstrated times of higher activity than the monocomponent counterpart.^11^

However, the controlled synthesis of ultrathin metal alloy nanosheets still remains a significant challenge due to the different reduction potentials of different components, various crystal structures, and a strong preference for close‐packing/stacking of metal atoms.^2^, ^12^ So the successful synthesis of ultrathin metal alloy nanosheets, even for only bimetallic case, was very limited.^13^, ^14^, ^15^, ^16^, ^17^, ^18^ Moreover, they were mostly noble‐metal‐based (e.g., PtCu,^14^, ^15^ PdAu,^16^ PdAg,^17^ and AuAg^18^), possibly because the noble metals are easier to reduce from salts, and the resultant nanostructures are stable at ambient condition. However, to the best of our knowledge, the preparation of nonnoble metal alloy nanosheets with single crystalline structure is not reported yet.

In this study, we synthesized single crystalline ultrathin Ni*_x_*Co_1−_ *_x_* alloy nanosheets arrays (ANSAs) with finely tunable compositions (Ni*_x_*Co_1−_ *_x_*, *x* = 0.9–0.5) by gently reducing a Ni*_x_*Co_1−_ *_x_*(OH)_2_ nanosheets array precursors under mild topochemical reduction conditions. The resultant Ni*_x_*Co_1−_ *_x_* alloy nanosheets showed face‐centered cubic (fcc) phase, with ultrathin (sub‐3 nm) single‐crystalline 2D structure, selectively exposed (111) facets, and optimized Ni:Co ratios, which exhibited much higher performance toward hydrazine oxidation reaction (HzOR) in DHFCs than the other elecrocatalysts, either Ni nanosheet array or Pt/C electrocatalyst. This demonstrated the feasibility of the topochemical conversion method, and also the advantage of alloy nanosheets.

As shown in **Scheme** **1**, Ni*_x_*Co_1−_ *_x_*(OH)_2_ nanosheets array was first synthesized on nickel foam substrate by slightly modifying our previous procedure.^19^ Nickel and cobalt salts were coprecipitated in an aqueous solution, induced by hexamethylene tetramine hydrolysis upon hydrothermal treatment at 100 °C. The elemental mapping of Ni_0.6_Co_0.4_(OH)_2_ nanosheets array (Figure S1, Supporting Information) revealed that Ni and Co elements were homogeneously distributed throughout the array. Then the as‐formed Ni*_x_*Co_1−_ *_x_*(OH)_2_ nanosheets were solvothermally reduced in alkaline ethylene glycol (EG) solution. Since most metal atoms have a strong preference for close‐packed crystalline structures, the solvothermal reduction must be well‐controlled in a moderate/mild condition to avoid self‐nucleation.

**Scheme 1:**
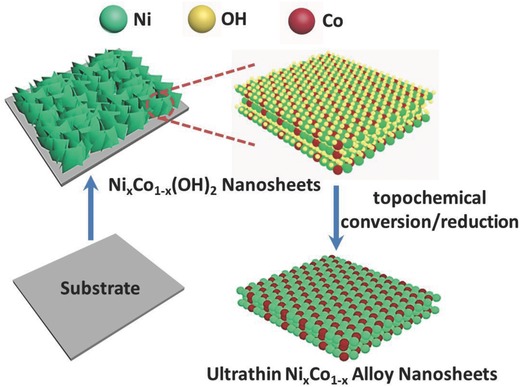
Schematically formation procedure of ultrathin Ni*_x_*Co_1−_ *_x_* alloy nanosheets through topochemical conversion/reduction from Ni*_x_*Co_1−_ *_x_*(OH)_2_ nanosheets grown on substrate.

Representative structure and morphology of the as‐prepared ultrathin Ni_0.6_Co_0.4_ alloy nanosheets array (Ni_0.6_Co_0.4_‐ANSA) was fully characterized by scanning electron microscopy (SEM), transmission electron microscopy (TEM), high‐resolution transmission electron microscopy (HRTEM), and scanning transmission electron microscopy (STEM). SEM image (**Figure** **1**A) of the Ni_0.6_Co_0.4_‐ANSA shows 3D porous structure with interconnected nanosheets (Figure 1B) growing vertically on the Ni foam substrate. It should be noted that most alloy nanosheets were kept on Ni substrate even after ultrasonication for 1 h, indicating the tight binding to the Ni substrate. As can be seen in the image (Figure 1B), the nanosheet is of micrometers in size. Figure 1C records the thickness of ≈2.5 nm on one laid nanosheet, demonstrating an ultrathin lamellar structure. The HRTEM image and electron diffraction pattern (Figure 1D,E) of a single layer nanosheet clearly reveal the fcc single‐crystal structure. Lattice fringes with an interplanar spacing of 0.21 nm are presented in the Figure 1F, corresponding to 1/3(422) fringes of fcc Ni_0.6_Co_0.4_ alloy nanosheet. During HRTEM characterization, focusing the electron beam on a small area will quickly damage these ultrathin nanosheets (see Figure S2, Supporting Information), indicating they are highly sensitive to electron beam irradiation. So electron exposures must be kept low enough during electron microscopy characterization to minimize irradiation damage to the alloy nanosheets. Elemental mapping using STEM on several overlapped nanosheets (Figure 1G) reveals both Ni and Co homogeneously distributed throughout the ultrathin nanosheets. The atomic ratio of Ni:Co = 59:41 and 58:42 of the alloy nanosheets were confirmed using energy‐dispersive X‐ray spectrometry (EDX) spectra and inductively coupled plasma (ICP) analysis (Figure S3 and Table S1 (Supporting Information)), respectively, which was roughly in accord with the feeding ratio of Ni:Co = 6:4. X‐ray photoelectron spectroscopy (XPS) further confirmed the ratio but also indicated the surface oxidation upon exposure of nanosheets in air for relatively long period (Figure S4, Supporting Information).

**Figure 1:**
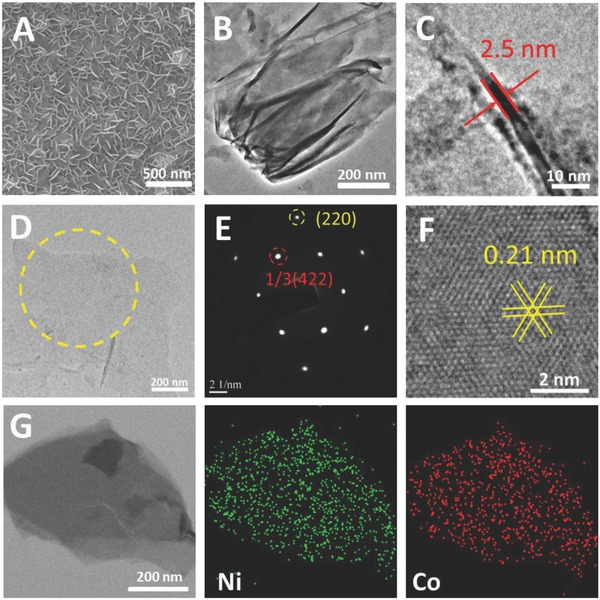
A) SEM image of as synthesized Ni_0.6_Co_0.4_‐ANSA. B) TEM image of a bunch of Ni_0.6_Co_0.4_ alloy nanosheets. C) The thickness of a vertically laid Ni_0.6_Co_0.4_ alloy nanosheet. D) HRTEM image of a single layer Ni_0.6_Co_0.4_ alloy nanosheet. E) Corresponding electron diffraction pattern and F) crystal lattices of the region marked in (D). G) STEM image and elemental mapping of Ni_0.6_Co_0.4_‐ANSs.

X‐ray diffraction (XRD) patterns of the typical sample Ni_0.6_Co_0.4_(OH)_2_ were collected to reveal the phase changes before and after solvothermal reduction, as shown in **Figure** **2**A. The Ni foam substrate shows only fcc phase Ni peaks before growth (blue line). Ni_0.6_Co_0.4_(OH)_2_ patterns emerged after the growth of hydroxide nanosheets arrays (β‐Ni(OH)_2_, JCPDS 14‐0117, red line). After reduction, the phase of hydroxide disappeared again because the Ni_0.6_Co_0.4_ alloy phase coincides with that of metallic Ni (data not shown). In order to confirm the reduction product was metallic Ni_0.6_Co_0.4_ alloy and exclude the influence of Ni substrate, we synthesized the Ni_0.6_Co_0.4_(OH)_2_ array samples on Cu foam substrate and did solvothermal reduction, too. Then the metallic Ni_0.6_Co_0.4_ alloy patterns shown just besides fcc Cu substrate, while that of hydroxides disappeared, indicating fully reduction from Ni_0.6_Co_0.4_(OH)_2_ to Ni_0.6_Co_0.4_ alloy.

**Figure 2:**
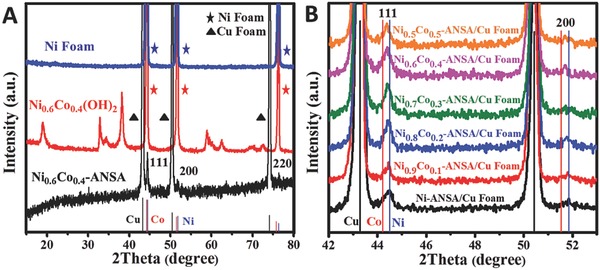
A) XRD pattern of Ni foam, the Ni_0.6_Co_0.4_(OH)_2_ nanosheets array grown on Ni foam and Ni_0.6_Co_0.4_‐ANSA grown on Cu foam. B) XRD patterns of Ni*_x_*Co_1−_ *_x_*‐ANSA with different atomic ratio on Cu foam. The three lines at the base show the standard XRD patterns of Cu, Co, and Ni.

Ni*_x_*Co_1−_ *_x_*(OH)_2_ (*x* = 0.9–0.5) nanosheets with varied Ni:Co atom ratios were synthesized by simply varying the original molar ratios of Ni:Co. The final Ni*_x_*Co_1−_ *_x_* alloy nanosheets after reduction inherit the original Ni:Co of hydroxides (see Table S1 in the Supporting Information for ICP analysis) and the sheet morphology (Figure S5, Supporting Information). The corresponding XRD patterns in the range of 42°–53° recorded on Ni*_x_*Co_1−_ *_x_*‐ANSA with different atomic ratios are shown in Figure 2B. It can be seen that the pure Ni‐NSA exhibits two Ni peaks at 44.5° and 51.8°, corresponding to (111) and (200) diffractions, respectively. While the two diffraction peaks of the ultrathin Ni*_x_*Co_1−_ *_x_* (Ni_0.9_Co_0.1_, Ni_0.8_Co_0.2_, Ni_0.7_Co_0.3_, Ni_0.6_Co_0.4_, and Ni_0.5_Co_0.5_) alloy nanosheets array gradually shift to lower 2*θ* angles compared to that of pure Ni‐NSA, indicating an expansion of the lattice. More Co leads to more shift to smaller angle, because Co atom (1.52 Å) is a little bigger than the size of Ni atom (1.49 Å). So the shift confirms the formation of Ni*_x_*Co_1−_ *_x_* alloy. It is found that when the Co element content reaches 50%, the obtained nanosheets become sparse and the array begins to fall down (Figure S6A, Supporting Information), and, after reduction, only Ni–Co alloy nanoparticles form, indicating changed growth and reduction behavior (Figure S6B–D, Supporting Information).

DHFC, which can directly transform chemical energy into electrical energy, has received more and more attention in recent years due to its high energy density and power density with great potential as clean power sources for future stationary and mobile applications.^20^ While the development of low‐cost electrodes for HzOR with superior catalytic activity would benefit a better performance of DHFC and accelerate its commercialization process. Here, we confirmed that Ni*_x_*Co_1−_ *_x_*‐ANSAs have intrinsic higher activity for HzOR using Ni nanosheets array (Ni‐NSA),^21^ Ni*_x_*Co_1−_ *_x_*(OH)_2_ nanosheets array, and 20 wt% Pt/C (loaded on Ni foam) as control samples in 3 mol L^−1^ KOH solution with 0.5 mol L^−1^ hydrazine. As shown in **Figure** **3**A, generally the ultrathin Ni*_x_*Co_1−_ *_x_*‐ANSAs exhibit a lower onset potential (*E*_on_) and faster current density increase as Co content increased, but the trend terminated as *x* = 0.5. Under optimized Ni:Co ratio, Ni_0.6_Co_0.4_‐ANSA shows the highest HzOR activity, which is in accord with the previous report.^11^ Figure 3B shows that the *E*_on_ of ultrathin Ni_0.6_Co_0.4_‐ANSA was 30 mV lower than that of Ni‐NSA, demonstrating a higher intrinsic activity toward hydrazine oxidation. Moreover, the Ni_0.6_Co_0.4_‐ANSA shows a significantly boosted current density enhancement for HzOR compared with other electrodes. At the same potential of −0.85 V, Ni_0.6_Co_0.4_‐ANSA electrode could afford a considerable current (292 mA cm^−2^), which is 1.41 and 2.41 times higher than Ni‐NSA (207 mA cm^−2^) and Pt/C electrode (122 mA cm^−2^), respectively. These results indicated that Ni_0.6_Co_0.4_‐ANSA exhibited ultrahigh electrocatalytic performance toward hydrazine oxidation. Electrochemical impedance spectroscopy shown in Figure 3C further supported the high electrocatalytic activity of the ultrathin Ni_0.6_Co_0.4_‐ANSA. The Nyquist plots and semicircles indicate that the HzOR on the four electrodes followed a similar mechanism but kinetically controlled. The ultrathin Ni_0.6_Co_0.4_‐ANSA possessed the smallest diameter of semicircle, which demonstrated it had the smallest charge transfer resistance (*R*_ct_) and the fastest kinetics for HzOR. In order to fully evaluate the actual performance of the ultrathin Ni_0.6_Co_0.4_‐ANSA, long‐term HzOR test was carried out at a constant potential of −0.88 V (Figure 3D). After 10000 s, the current density could maintain 84.8% of its initial value. In contrast, the current density of Pt/C decreased to below 50%, revealing the superb stability of Ni_0.6_Co_0.4_‐ANSA. Figure S7 (Supporting Information) showed the SEM image of Ni_0.6_Co_0.4_‐ANSA after long‐term stability test, the well‐preserved morphology confirmed the structural stability.

**Figure 3:**
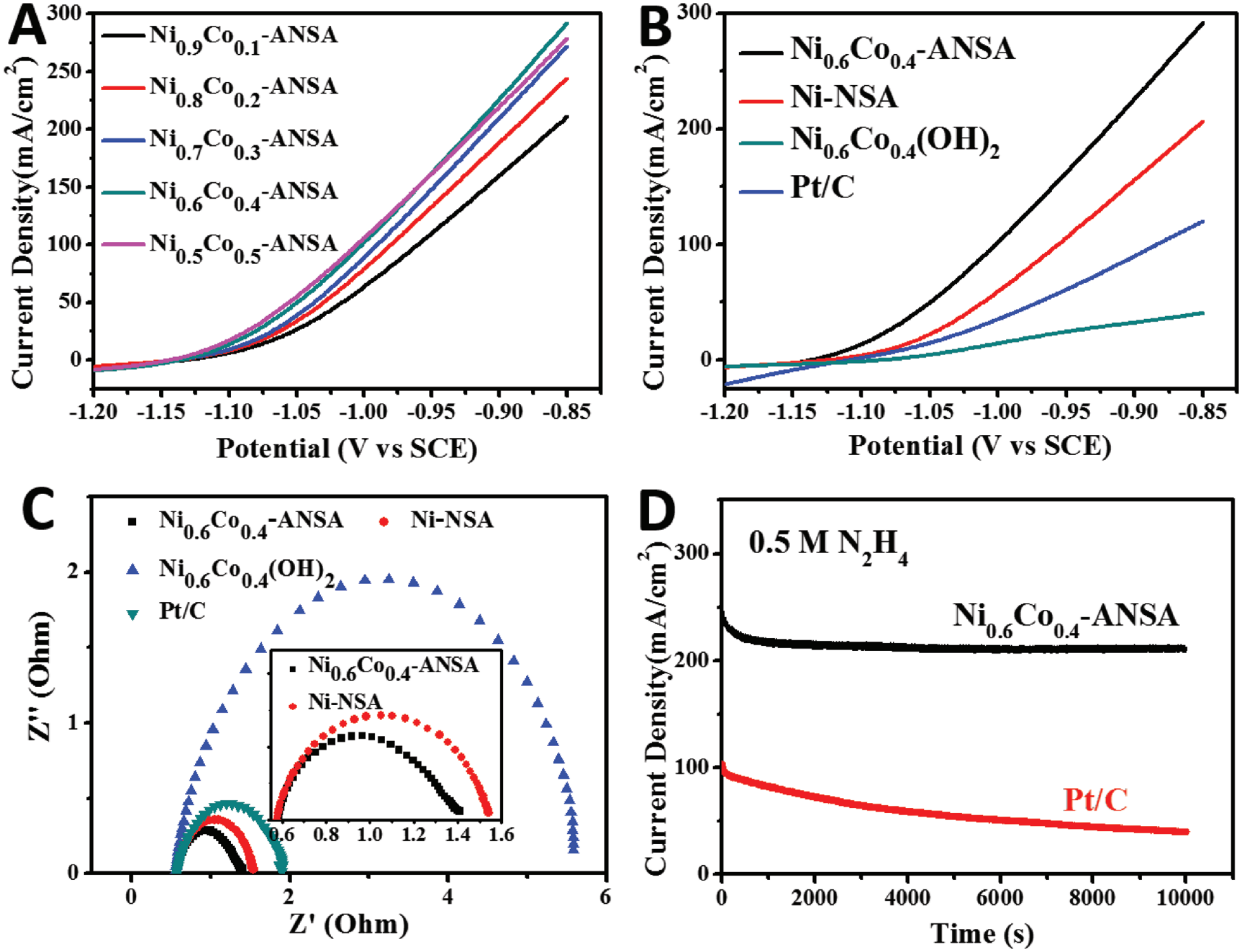
A) HzOR polarization curves of Ni_0.9_Co_0.1_‐ANSA, Ni_0.8_Co_0.2_‐ANSA, Ni_0.7_Co_0.3_‐ANSA, Ni_0.6_Co_0.4_‐ANSA, and Ni_0.5_Co_0.5_‐ANSA in 3 m KOH solution with 0.5 m hydrazine. B) LSV curves of Ni_0.6_Co_0.4_‐ANSA, Ni‐NSA, Ni_0.6_Co_0.4_(OH)_2_ nanosheets array, and 20 wt% Pt/C (loaded on Ni foam), demonstrating that the ultrathin Ni_0.6_Co_0.4_‐ANSA afford much faster current increases and lower onset potential than the other electrodes. C) Nyquist plots of the four electrodes, indicating the Ni_0.6_Co_0.4_‐ANSA possesses a much smaller charge‐transfer resistance. D) Stability testing of the Ni_0.6_Co_0.4_‐ANSA and 20 wt% Pt/C, demonstrating a good stability.

Such overwhelming electrocatalytic performance of the ultrathin Ni_0.6_Co_0.4_‐ANSA is ascribed to the extremely high intrinsic activity toward hydrazine oxidation,^11^, ^22^ and the ultrahigh surface areas and a plethora of unsaturated atoms (i.e., surface atoms, step/corner atoms) induced by the ultrathin 2D nanostructures. Those dangling atoms could act as highly active electrocatalytic sites. The electrochemical double‐layer capacitance (EDLC) of the electrodes could be used to estimate the effective electrochemically active surface area of the solid–liquid interfaces.^9^, ^23^ As shown in Figure S8 (Supporting Information), the EDLC values of Ni_0.6_Co_0.4_‐ANSA, Ni‐NSA, and Pt/C were 34.8, 29.9, and 12.5 mF cm^−2^, respectively, which indicated that Ni_0.6_Co_0.4_‐ANSA had the largest effective electrochemical area. Another important merit is that the tight binding to the electroconductive substrate and 3D open porous structure make all the ultrathin nanosheets chemically accessible for reactant and meanwhile keep good electric connect to external circuit.

As a demonstration for practical application, the direct liquid N_2_H_4_/H_2_O_2_ fuel cell (DHHPFC) assembled by the ultrathin Ni_0.6_Co_0.4_‐ANSA and commercial Pt/C catalyst as the anode and cathode, respectively, was demonstrated in **Figure** **4**. Compared to N_2_H_4_/O_2_ fuel cell, the DHHPFC has much higher theoretical open circuit voltage and power density due to the H_2_O_2_ dramatically improving reaction kinetics.^24^, ^25^ The schematic diagram (Figure 4A) showed the electrode reactions and working mechanism of the DHHPFC. When N_2_H_4_ was oxidated to N_2_, H_2_O_2_ was reduced to H_2_O. As the electrons transferring from anode to cathode, K^+^, as the main charge carrier, moved in an opposite direction through a Nafion membrane.^25^ The overall reaction could be written as follows

(1) $$N_{2}H_{4}+\mathrm{2KOH}+{\mathrm{2H}}_{2}O_{2}+H_{2}{\mathrm{SO}}_{4}\to N_{2}↑+\;\;\;{\mathrm{6H}}_{2}O+K_{2}{\mathrm{SO}}_{4}$$

**Figure 4:**
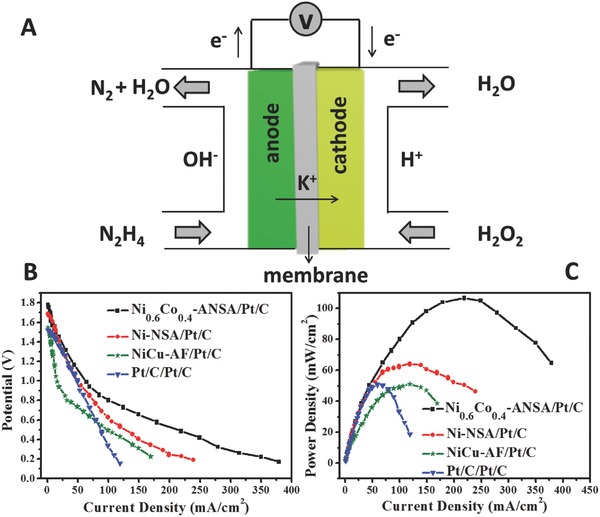
A) Schematic diagram of a DHHPFC. B) The current–voltage (*I*–*V*) characterization of DHHPFCs assembled using Ni_0.6_Co_0.4_‐ANSA, Ni‐NSA, NiCu‐AF, 40 wt% Pt/C as the anode, and Pt/C as the cathode, respectively (cell temperature: 80 °C). C) The current–power density (*I*–*P*) performance of the above four fuel cells. Note: The mass loadings of Ni_0.6_Co_0.4_‐ANSA, Ni‐NSA, NiCu film, and 40 wt% Pt/C were 1.4, 1.18, 2.2, and 2 mg, respectively. All the cathodes were 40 wt% Pt/C.

As comparisons, Ni‐NSA, reported NiCu alloy film (NiCu‐AF),^26^ 40 wt% Pt/C, IrO_2_, and RuO_2_ were also tested. The different current–voltage (*I*–*V*) characteristics were observed in Figure 4B and Figure S9A (Supporting Information) when using anodes with different compositions. The open‐circuit voltage of the cell using Ni_0.6_Co_0.4_‐ANSA (1.781 V) was higher than that of the other anodes, indicating the highest intrinsic activity. Moreover, the maximum power density (107.1 mW cm^−2^) of Ni_0.6_Co_0.4_‐ANSA (Figure 4C and Figure S9B (Supporting Information)) was 1.66, 2.08, 2.09, 2.48, and 2.17 times higher than that of assembled with Ni‐NSA (64.5 mW cm^−2^), NiCu‐AF (51.5 mW cm^−2^), commercial Pt/C (51.1 mW cm^−2^), IrO_2_ (43.2 mW cm^−2^), and RuO_2_ (49.4 mW cm^−2^), respectively. The loading‐mass normalized data were compared in Figure S10 (Supporting Information): the mass power density of Ni_0.6_Co_0.4_‐ANSA was 76.6 mW mg^−1^, which was 1.40 and 3.03 times higher than that of Ni‐NSA and commercial Pt/C. The above data fully demonstrated the benefit by using NiCo ultrathin nanosheet arrays.

In conclusion, by a facile solvothermal reduction method, ultrathin nickel–cobalt alloy nanosheets array (Ni*_x_*Co_1−_ *_x_*‐ANSA) was achieved. The ultrathin Ni_0.6_Co_0.4_ alloy nanosheets with thickness of 2.5 nm exposed single‐crystalline lamellar structure. With ultrathin 2D structure, high surface area, tight binding to the electroconductive substrate, and high intrinsic activity, the prepared ultrathin Ni_0.6_Co_0.4_‐ANSA was demonstrated as a highly effective electrocatalyst for HzOR in DHFC with activity and stability much higher than Pt/C or Ni nanoarray. Such alloy ultrathin nanostructure synthesis strategy should be extendable to other nonprecious metal alloys and provides wide possibility for their composition and structure tuning.

## Experimental Section

*Synthesis of Ni_x_Co_1−x_(OH)_2_ Nanosheets Array*: Ni*_x_*Co_1−_ *_x_*(OH)_2_ nanosheets array were fabricated by slightly modifying the previous methodology.^19^ In a typical synthesis of Ni_0.6_Co_0.4_(OH)_2_ nanosheets array, Ni(NO_3_)_2_·6H_2_O (0.87 g, 3 mmol), Co(NO_3_)_2_·6H_2_O (0.58 g, 2 mmol), and hexamethylenetetramine (1.40 g, 10 mmol) were mixed in 38 mL distilled water at room temperature and stirred to form a clear solution. Nickel foam (about 4 cm × 2 cm) was carefully cleaned with concentrated HCl solution (37 wt%) in an ultrasound bath for 30 s in order to remove the surface oxide layer. And then rinsed with deionized water and absolute ethanol to ensure the surface of the Ni foam was well cleaned. The aqueous solution and the Ni foam were transferred to a 40 mL Teflon‐lined stainless‐steel autoclave, which was sealed, maintained at 100 °C for 12 h, and then allowed to cool to room temperature naturally. The thin nanosheets on the metal substrate were washed several times with distilled water and ethanol with the assistance of ultrasonication, and dried at 80 °C for 6 h. Other Ni*_x_*Co_1−_ *_x_*(OH)_2_ nanosheets array with different molar ratios could be synthesized by following similar process as Ni_0.6_Co_0.4_(OH)_2_ nanosheets array.

*Synthesis of Ni_x_Co_1−x_‐ANSA*: Ni*_x_*Co_1−_ *_x_*‐ANSA was prepared by topochemical converting/reducing Ni*_x_*Co_1−_ *_x_*(OH)_2_ nanosheets array precursors. In a typical procedure of Ni_0.6_Co_0.4_‐ANSA, NaOH (1 g) and EG (36 mL) were put into a 40 mL Teflon‐lined stainless‐steel autoclave, and stirred until the solid dissolved. Then Ni_0.6_Co_0.4_(OH)_2_ nanosheets array was transferred to the Teflon‐lined stainless‐steel autoclave, maintained at 160 °C for 12 h, and allowed to cool to room temperature naturally. The cleaning treatment of Ni_0.6_Co_0.4_‐ANSA was similar to those of Ni*_x_*Co_1−_ *_x_*(OH)_2_ nanosheets array, but the drying process must be carried out in the environment without oxygen to avoid oxidation. And other Ni*_x_*Co_1−_ *_x_*‐ANSA with different molar ratios could be also synthesized by the similar process used for preparing Ni_0.6_Co_0.4_‐ANSA.

*Materials Characterizations*: XRD patterns were collected on an X‐ray diffractometer (Rigaku D/max 2500) at a scan rate of 5 (°) min^−1^, recorded with 2*θ* ranging from 15° to 90°. Elemental analysis was investigated by inductively coupled plasma optical emission spectrometry (Thermo Scientific iCAP 6300). XPS measurements were performed using a Thermo Electron ESCALAB 250 instrument (Al Kα, 200 W). The morphologies of as‐synthesized samples were monitored using SEM (Zeiss Supra 55) and HRTEM (JEOL, JEM‐2100, 200 kV) equipped with an EDX instrument. Elemental mapping were obtained on a JEOL 2100 high‐resolution TEM system.

*Electrochemical Measurements*: All electrochemical tests of the HzOR performance were performed in a standard three‐electrode electrochemical cell with Pt foil (1 cm^2^) and saturated calomel electrode used as counter electrode and reference electrode at room temperature (≈20 °C) using a CHI 660D (Chenghua, Shanghai) electrochemical workstation. 1 cm × 1 cm area of Ni_0.6_Co_0.4_‐ANSA, Ni‐NSA, and Ni_0.6_Co_0.4_(OH)_2_ nanosheets array were used directly as working electrodes while 1 mg 20 wt% Pt/C were loaded on Ni foam substrate as working electrodes. It should be noted that the loading amounts of Pt/C were more than that of Ni_0.6_Co_0.4_ alloy nanosheets (0.7 mg cm^−2^). In the hydrazine oxidation reaction, cyclic voltammetry and linear sweep voltammetry (LSV) with scan rate of 5 mV s^−1^ were conducted in 3 m KOH solution containing 0.5 m hydrazine. AC impedance measurements were carried out in the same configuration at −1.05 V from 10^−1^ to 10^5^ Hz with an AC voltage of 5 mV. The stability tests were operated at high overpotentials to achieve high initial current densities.

The DHHPFC was tested in a stable operating condition. The aqueous solution containing 20% N_2_H_4_ and 4 m KOH was added into the anode side at a flow rate of 5 mL min^−1^ by a peristaltic pump and silicone tubes. Meanwhile, the liquid feed in the cathode side was the acidic solution containing 20% H_2_O_2_ and 0.5 m H_2_SO_4_ with a same flow rate by another peristaltic pump. Nafion 115 membrane which was treated to remove organic contaminants and heavy metal ions was used as the solid electrolyte. The cell temperature was controlled via a temperature controller and monitored by thermocouples buried in the graphite blocks. The steady state polarization curves were recorded by an automatic electric Load (PLZ 70UA, Japan). The pure Ni‐NSA, NiCu‐AF, and commercial Pt/C film were used as the anodes for comparison purposes. 40 wt% Pt/C loaded on carbon paper was used as the cathode in all cells.

## Supporting information

As a service to our authors and readers, this journal provides supporting information supplied by the authors. Such materials are peer reviewed and may be re‐organized for online delivery, but are not copy‐edited or typeset. Technical support issues arising from supporting information (other than missing files) should be addressed to the authors.

Supplementary
