# Supplementary material for: Single Crystalline Ultrathin Nickel–Cobalt Alloy Nanosheets Array for Direct Hydrazine Fuel Cells
Source: Adv Sci (Weinh). 2016 Dec 20;4(3):1600179. doi: 10.1002/advs.201600179 (PMC5357988; doi:10.1002/advs.201600179)
Supplement: Supplementary file 1 — Supplementary [file ADVS-4-na-s001.pdf]

Copyright WILEY-VCH Verlag GmbH & Co. KGaA, 69469 Weinheim, Germany, 2016.

ADVANCED SCIENCE Open Access

Supporting Information

for *Adv. Sci.,* DOI: 10.1002/advs.201600179

Single Crystalline Ultrathin Nickel–Cobalt Alloy Nanosheets Array for Direct Hydrazine Fuel Cells

*Guang Feng, Yun Kuang, Pengsong Li, Nana Han, Ming Sun, Guoxin Zhang, and Xiaoming Sun**

Supporting Information

Single Crystalline Ultrathin Nickel-Cobalt Alloy Nanosheets Array for Direct Hydrazine Fuel Cells

Guang Feng, Yun Kuang, Pengsong Li, Nana Han, Ming Sun, Guoxin Zhang, and *Xiaoming Sun**

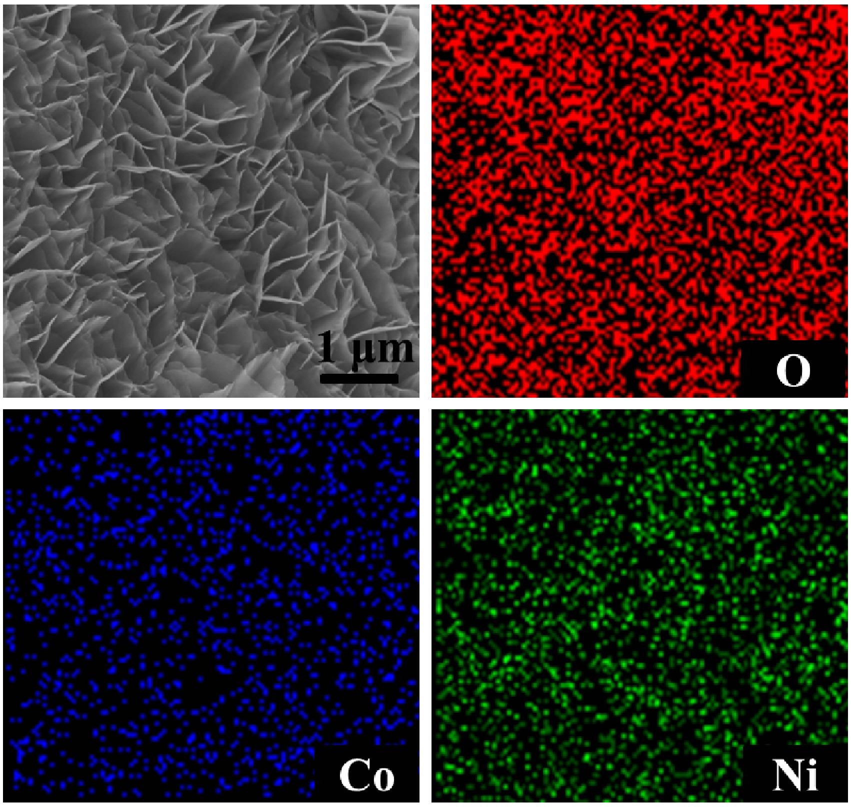

Figure S1. Elemental mapping of the $Ni_{0.6}Co_{0.4}(OH)_2$ nanosheets array. Red: oxygen, Blue: cobalt, Green: nickel, revealing that Ni and Co elements were homogeneously distributed throughout the nickel-cobalt hydroxide array.

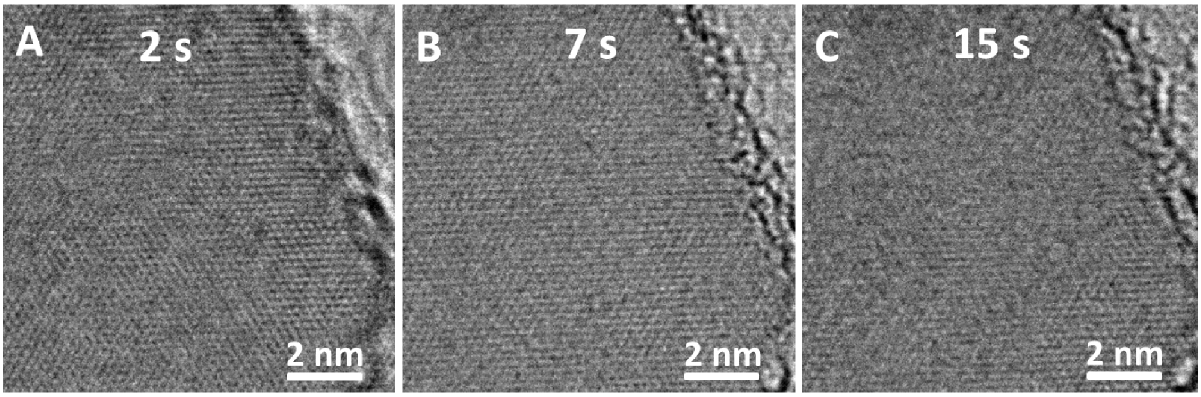

Figure S2. Crystal lattices images of $Ni_{0.6}Co_{0.4}$ alloy nanosheets after beam exposure for different time: (A) 2 s, (B) 7 s, (C) 15 s. It can be seen that ultrathin nanosheets are highly sensitive to irradiation and longtime exposure under electron beams would damage the nanosheets, so the electron exposure must be kept as low as possible during electron microscopy characterization to minimize irradiation damage.

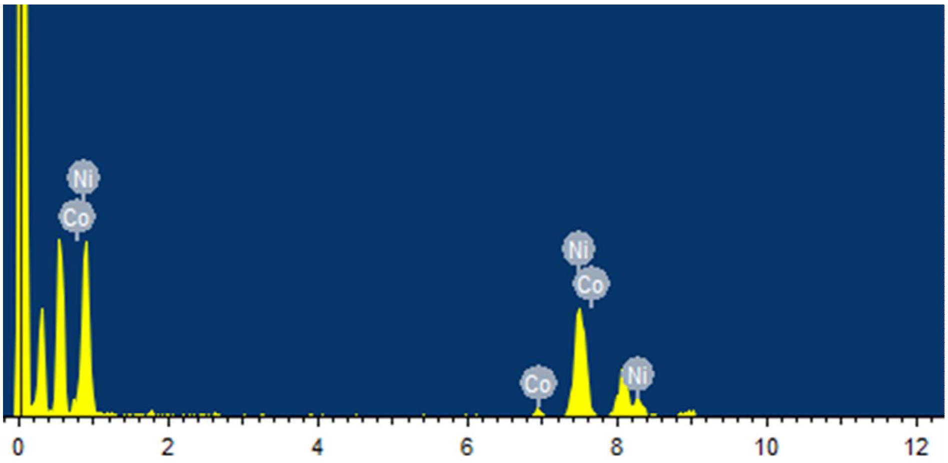

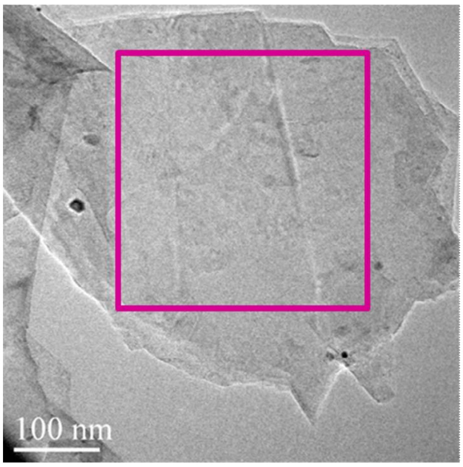

Sample	$Ni_{0.6}Co_{0.4}$ alloy nanosheets
Ni atom percent (%)	58.95
Co atom percent (%)	41.05
Ni:Co atom ratio	≈ 0.59:0.41

Figure S3. EDX spectra of the region marked in the TEM image of several overlapped $Ni_{0.6}Co_{0.4}$ alloy nanosheets, demonstrating a local Ni:Co atom ratio in the nanosheets was ≈ 0.59:0.41, which agreed with the ICP analysis of the whole alloy sample grown on Cu substrate.

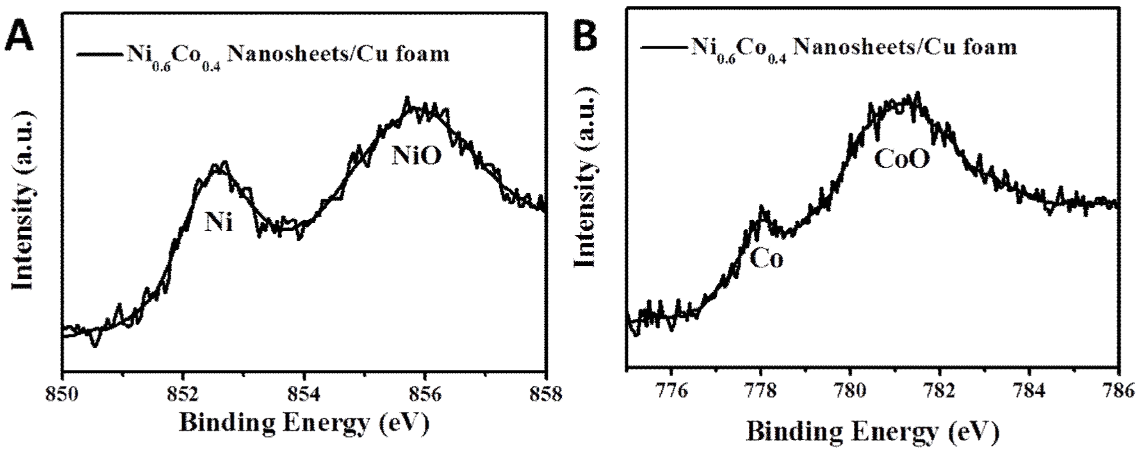

Figure S4. XPS spectra of $Ni_{0.6}Co_{0.4}$-ANSA loaded on Cu foam. Since the surface was easy to be oxidized in ambient atmosphere, the nanosheets should be stored in the environment without oxygen.

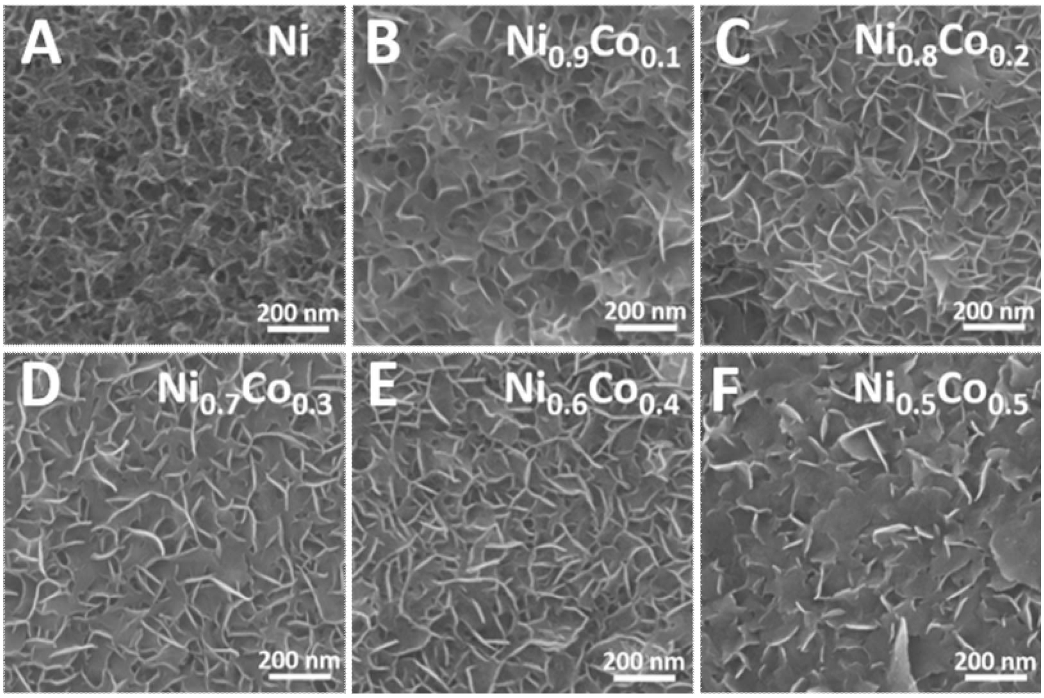

Figure S5. SEM images of Ni_XCo_{1-X}-ANSA with different feeding ratio: (A) pure Ni-NSA, (B) $Ni_{0.9}Co_{0.1}$-ANSA, (C) $Ni_{0.8}Co_{0.2}$-ANSA, (D) $Ni_{0.7}Co_{0.3}$-ANSA, (E) $Ni_{0.6}Co_{0.4}$-ANSA, (F) $Ni_{0.5}Co_{0.5}$-ANSA.

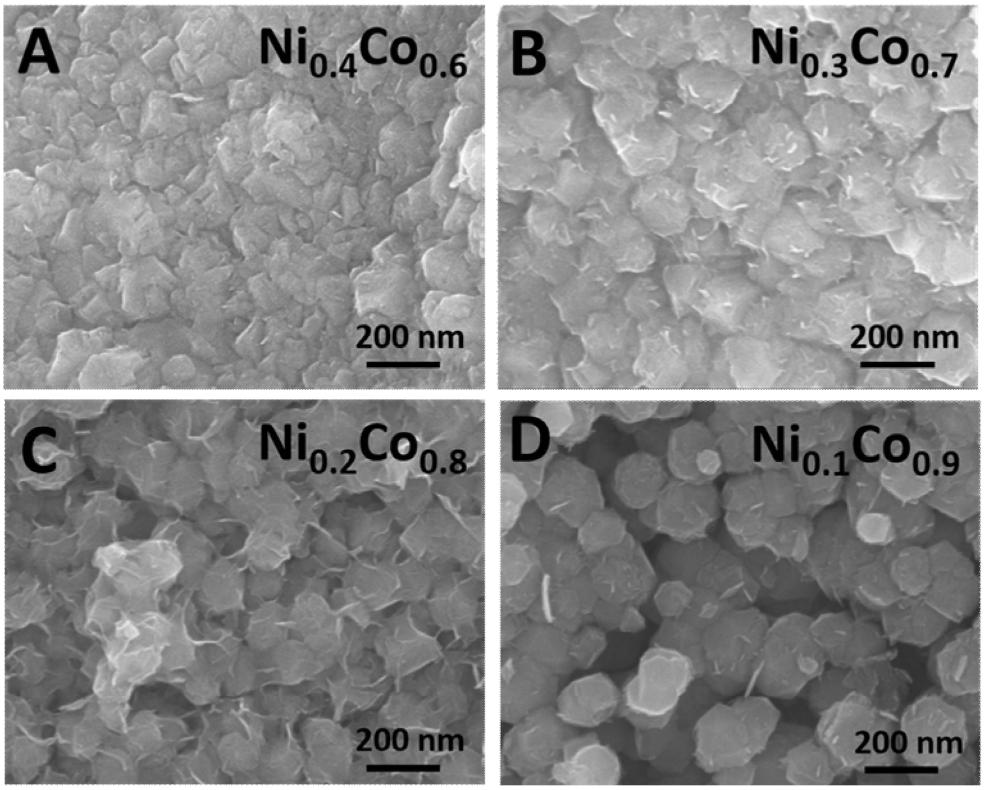

Figure S6. SEM images of Ni_xCo_{1-x} alloy with other feeding ratios of Ni:Co. (A) $Ni_{0.4}Co_{0.6}$, (B) $Ni_{0.3}Co_{0.7}$, (C) $Ni_{0.2}Co_{0.8}$, (D) $Ni_{0.1}Co_{0.9}$.

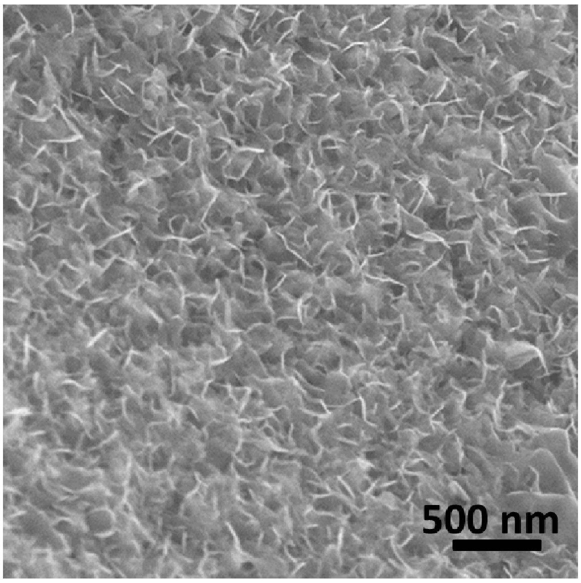

Figure S7. SEM image of $Ni_{0.6}Co_{0.4}$-ANSA after a long-term stability test of HzOR, demonstrating the ultrathin morphology of the nanosheets was well-preserved.

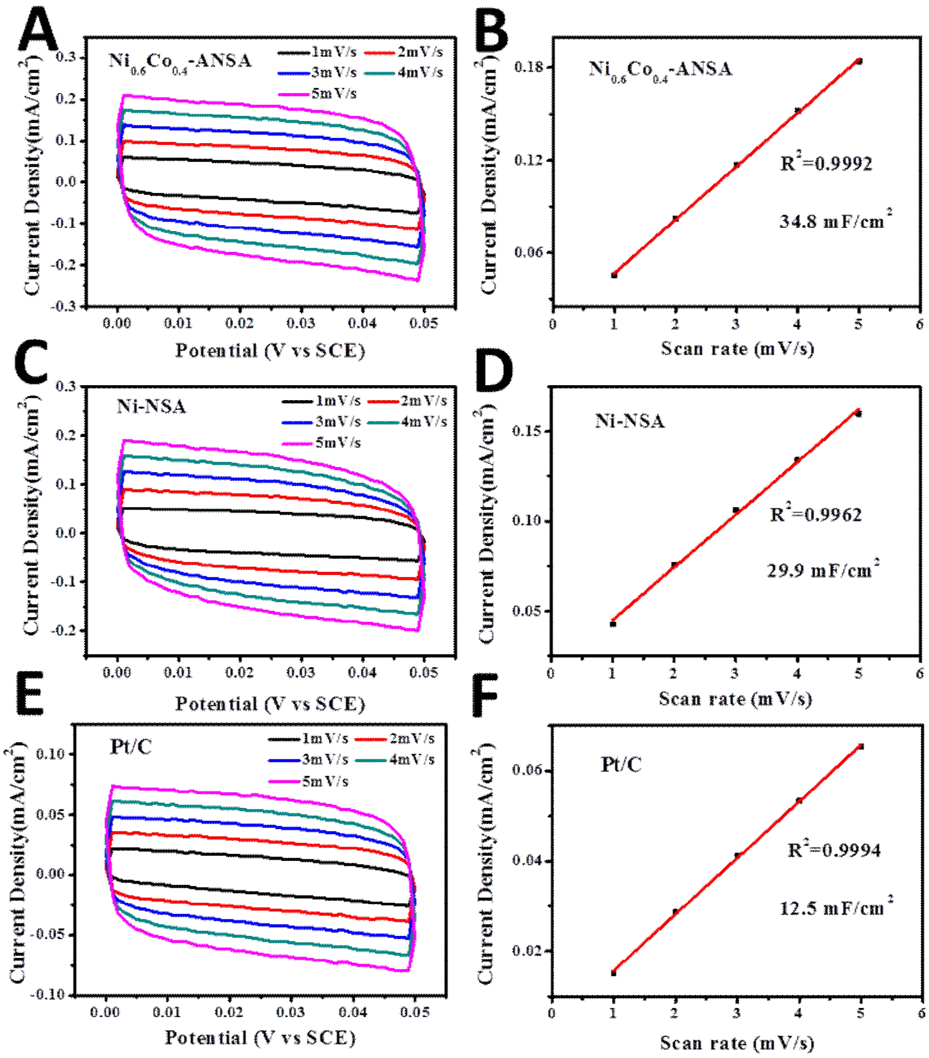

Figure S8. The electrochemical double-layer capacitance (EDLC) measurements and the corresponding current density-scan rates curves of $Ni_{0.6}Co_{0.4}$-ANSA (A and B), Ni-NSA (C and D) and Pt/C (E and D) at various scan rates from 0 V to 0.05 V.

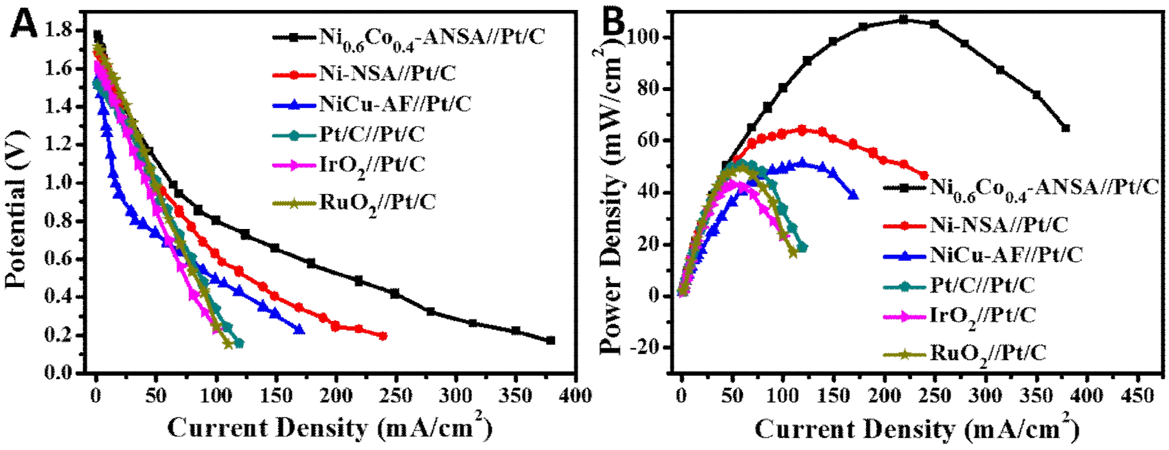

Figure S9. The performance of DHHPFCs assembled using $Ni_{0.6}Co_{0.4}$-ANSA, Ni-NSA, NiCu -AF, 40 wt% Pt/C, IrO_2, RuO_2 as the anode, and Pt/C as cathode, respectively. (cell temperature: 80 °C). (A) The current-voltage curves, (B) The current-power density curves. Note: The mass loadings of $Ni_{0.6}Co_{0.4}$-ANSA, Ni-NSA, NiCu-AF, 40 wt% Pt/C, IrO_2, and RuO_2 were 1.4 mg, 1.18 mg, 2.2 mg, 2 mg, 2mg, and 2 mg, respectively. All the cathodes were 40 wt% Pt/C.

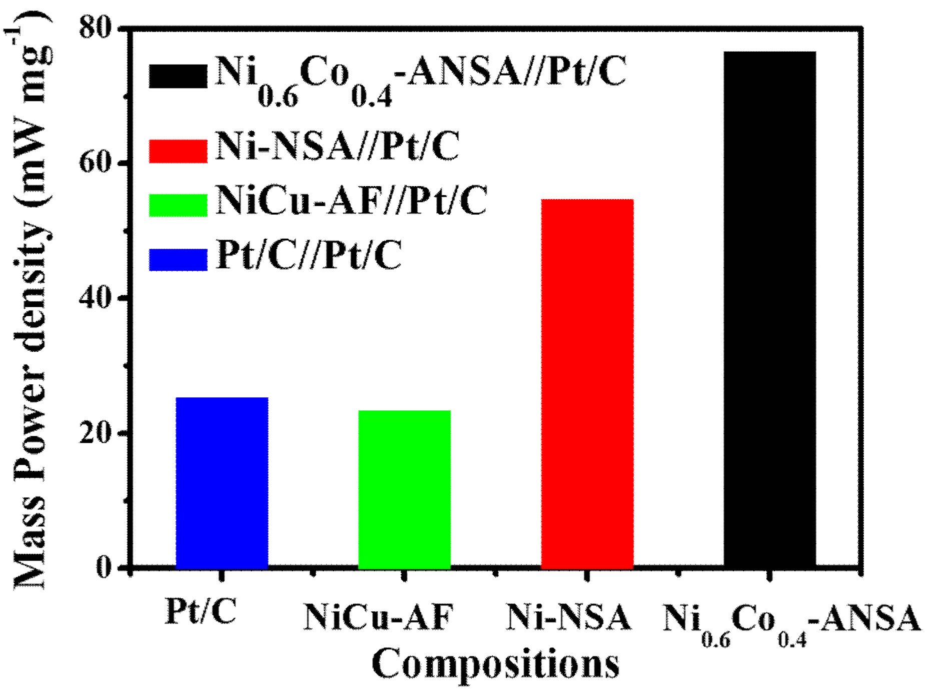

Figure S10. Quantitative comparisons of the mass power density of the four cells, assembled by $Ni_{0.6}Co_{0.4}$-ANSA, Ni-NSA, NiCu-AF and Pt/C as the anodes, respectively.

Sample	Composition from Synthesis/ at%	Composition by ICP Measurement/ at%
Ni_xCo_{1-x}	**0.90 : 0.10**	**0.938 : 0.062**
	**0.80 : 0.20**	**0.806 : 0.194**
	**0.70 : 0.30**	**0.701 : 0.299**
	**0.60 : 0.40**	**0.577 : 0.423**
	**0.50 : 0.50**	**0.444 : 0.556**

Table S1. The ICP measurements of Ni_xCo_{1-x}-ANSA with various atom ratio ($Ni_{0.9}Co_{0.1}$, $Ni_{0.8}Co_{0.2}$, $Ni_{0.7}Co_{0.3}$, $Ni_{0.6}Co_{0.4}$, and $Ni_{0.5}Co_{0.5}$).
